# The Future of Artificial Intelligence in Surgery

**DOI:** 10.7759/cureus.63699

**Published:** 2024-07-02

**Authors:** Allan Hamilton

**Affiliations:** 1 Artificial Intelligence Division for Simulation, Education, and Training, University of Arizona Health Sciences, Tucson, USA

**Keywords:** assessment of surgical proficiency, space exploration, operative anatomic overlay, augmented reality, diagnostic decision support systems, image-guided surgery, operative support systems, machine learning (ml), artificial intelligence (ai)

## Abstract

Until recently, innovations in surgery were largely represented by extensions or augmentations of the surgeon’s perception. This includes advancements such as the operating microscope, tumor fluorescence, intraoperative ultrasound, and minimally invasive surgical instrumentation. However, introducing artificial intelligence (AI) into the surgical disciplines represents a transformational event. Not only does AI contribute substantively to enhancing a surgeon’s perception with such methodologies as three-dimensional anatomic overlays with augmented reality, AI-improved visualization for tumor resection, and AI-formatted endoscopic and robotic surgery guidance. What truly makes AI so different is that it also provides ways to augment the surgeon’s cognition. By analyzing enormous databases, AI can offer new insights that can transform the operative environment in several ways. It can enable preoperative risk assessment and allow a better selection of candidates for procedures such as organ transplantation. AI can also increase the efficiency and throughput of operating rooms and staff and coordinate the utilization of critical resources such as intensive care unit beds and ventilators. Furthermore, AI is revolutionizing intraoperative guidance, improving the detection of cancers, permitting endovascular navigation, and ensuring the reduction in collateral damage to adjacent tissues during surgery (e.g., identification of parathyroid glands during thyroidectomy). AI is also transforming how we evaluate and assess surgical proficiency and trainees in postgraduate programs. It offers the potential for multiple, serial evaluations, using various scoring systems while remaining free from the biases that can plague human supervisors. The future of AI-driven surgery holds promising trends, including the globalization of surgical education, the miniaturization of instrumentation, and the increasing success of autonomous surgical robots. These advancements raise the prospect of deploying fully autonomous surgical robots in the near future into challenging environments such as the battlefield, disaster areas, and even extraplanetary exploration. In light of these transformative developments, it is clear that the future of surgery will belong to those who can most readily embrace and harness the power of AI.

## Introduction and background

From the beginning of medical history dating back to the Babylonian Code of Hammurabi (1750 BCE) and the Egyptian Smith Papyrus (ca. 1600-1500 BCE), progress in the field of surgery has been shaped by the contributions made in the realms of anatomy, pharmacology, and technology [1]. Advances in the field rested upon the substantial but finite powers of the human surgeon who stood at the center of the operating suite, their hands and eyes assuming command of the surgical field. While there have been tremendous inroads across the fronts of dozens of surgical disciplines, we can conveniently group most of them under the principle of enhancing or expanding the breadth of the human umwelt.

Umwelt is a German term first coined by Estonian biologist Jakob Johann von Freiherr Uexküll (1864-1944). He used the term “the world around us” or “the self world.” It was his way of describing the highly individual perceptual space within which each human, each organism, establishes a functional identity through its sensorium. It is also the physiological space within which the individual lives and acts [2]. To put this into surgical perspective, surgeons can only visualize a surgical field with their eyes. We can extend the capacity of the surgeons’ individual, neurophysiologically defined space [3] by providing them, for example, with loupe magnification, an operative microscope, or even an overlay of anatomic structures derived from the patient’s specific MRI studies. We can even enhance the surgeon’s visual sensitivity beyond visible light by adding tissue markers or dyes that will fluoresce in the appropriate ultraviolet or infrared spectra [4]. While it might seem, at first glance, that we have dramatically broadened the surgeon’s umwelt, we have not. We have effectively created an engineering infrastructure that allows us to move what lies beyond the umwelt and include it within it. For example, we did not alter the surgeon’s vision to gain access to previously invisible segments of the electromagnetic spectrum (EMS) [5]. Nothing about the physical characteristics of these segments of the EMS was altered; those segments remain invisible to the unaided eye.

**Figure 1:**
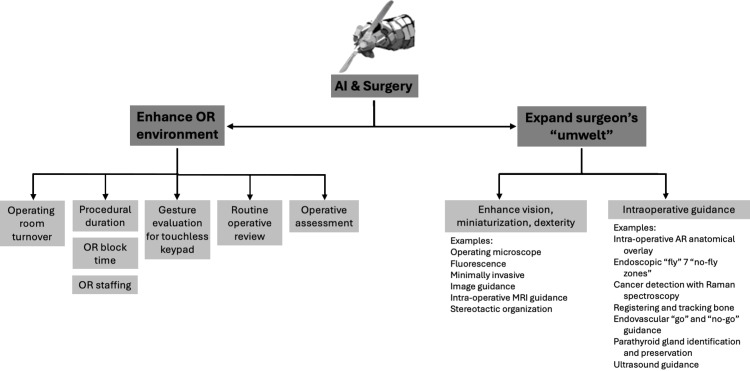
The two ways AI can work in surgery. A schematic showing the two ways in which AI can work in surgery. The first way is depicted here, and the second way is by expanding the surgeon’s “umwelt” (Figure 2). It shows that AI can work effectively by expanding the surgeon’s cognition, that is, by providing decision support by expanding the availability and analysis of data that would otherwise be unavailable or inaccessible to the surgeon. AI-derived management will also substantially increase the productivity of the operating room. Author’s own illustration. AI: artificial intelligence; OR: operating room; MRI: magnetic resonance imaging; AR: augmented reality

Under nighttime conditions, soldiers operate efficiently with night vision goggles. Their motto is, “We own the night.” The goggles capture deficient levels of ambient photons still present at night but in quantities so low as not to be visible to the human eye. The photons are amplified thousands of times by a microchannel plate component. The amplified photons are then accelerated toward a phosphor screen or viewfinder, causing the phosphor to glow and emit visible light. The brightness of the glow corresponds to the number of amplified electrons impinging on the phosphor screen, creating a visible image from the initially dim scene. We transformed the darkness not by altering the properties of the human eye but rather by the photonic quality of the darkness itself [6]. In an analogous fashion, engineering technology has allowed surgeons to maneuver surgical instrumentation in more limited surgical fields with the introduction of laparoscopic and robotic instrumentation. But every one of these advances has relied upon the extant umwelt of the operator (Figure 1).

**Figure 2:**
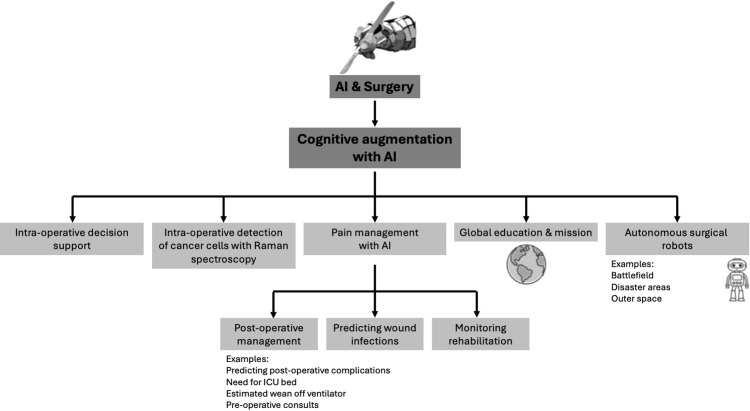
How AI can work in surgery by expanding the surgeon’s “umwelt.” A schematic showing the second way in which AI can work in surgery. The method shown here is by effectively expanding the surgeon’s “umwelt.” By expanding the surgeon’s ability to perceive, it makes the surgeon more effective. Author’s own illustration*.* AI: artificial intelligence; ICU: intensive care unit

A new era dawned in the 21st century with the integration of artificial intelligence (AI) into surgical support systems, heralding a profound wave of surgical innovation that surpassed human perception. In the past, surgical technology catered to the surgeon’s senses, but the new form that innovation has taken can no longer be satisfied with enhancing perception but, instead, strives to improve cognition [7]. For the first time in medical history, the progress unfolding now might come at the cost of the surgeon’s judgment (Figure 2). In the past, all progress was intricately tied to the surgeon’s expertise, but with the emergence of AI, advancements in surgical outcomes may no longer rely solely on technique. They may stem from an analysis of intricate databases, diagnostic decision support systems, predictive analytics, and enhancements in surgical instrumentation and robotic manipulation that surpass human physical capabilities.

Furthermore, we must not limit our vision of AI to merely bolstering surgical capabilities within our hospitals and clinics. We must envision the tangible benefit of applying AI to support autonomous surgical robots capable of operating in terrestrial environments where climate, disaster, or war have rendered surgical capacity unsustainable. AI will also be able to address the need for surgical intervention in extraterrestrial scenarios where insurmountable distances will place patients beyond the reach of a human surgeon.

## Review

Past history

Postoperative Complications and Management

Postoperative complications account for more than a doubling of the mortality and the costs of surgery. In a large single cohort study conducted by the University of Florida at Gainesville (UFG), the medical records of over 50,000 surgical patients who had undergone major surgical procedures were employed to validate an automated analytic framework designed to probabilistically forecast a patient’s risk of developing any one of eight common severe life-major complications. The complications included sepsis, deep venous thromboembolism, wound infection, acute kidney injury, a stay in the intensive care unit (ICU) after surgery that lasted for more than 48 hours, the need for respiratory support on a ventilator for more than 48 hours, neurologic impairment, cardiovascular complications, and death occurring during the postoperative follow-up period of 24 months. The UFG model (named MySurgeryRisk) calculated the risk of eight different complications with the area under the curve (AUC) ranging between 0.82 and 0.94 (99% confidence intervals (CIs) = 0.81-0.94). The model also calculated the actuarial risk for death at 1, 3, 6, 12, and 24 months, with AUC values ranging between 0.77 and 0.83 (99% CI = 0.76-0.85). In short, the UFG algorithm could provide a dynamic, invaluable adjunct to the surgeon’s judgment on the severity of postoperative morbidity and mortality the patient might incur due to surgery [8]. This kind of “probabilistic surgical reflection” was alluded to earlier in the discussion about AI’s impact on surgery being considerably more aligned with addressing cognitive issues than simply restricting itself to technical ones. As William J. Mayo astutely observed in 1921: “That which can be foreseen can be prevented” [9].

Area Under the Curve Calculation and Receiver Operating Characteristic

**Figure 3:**
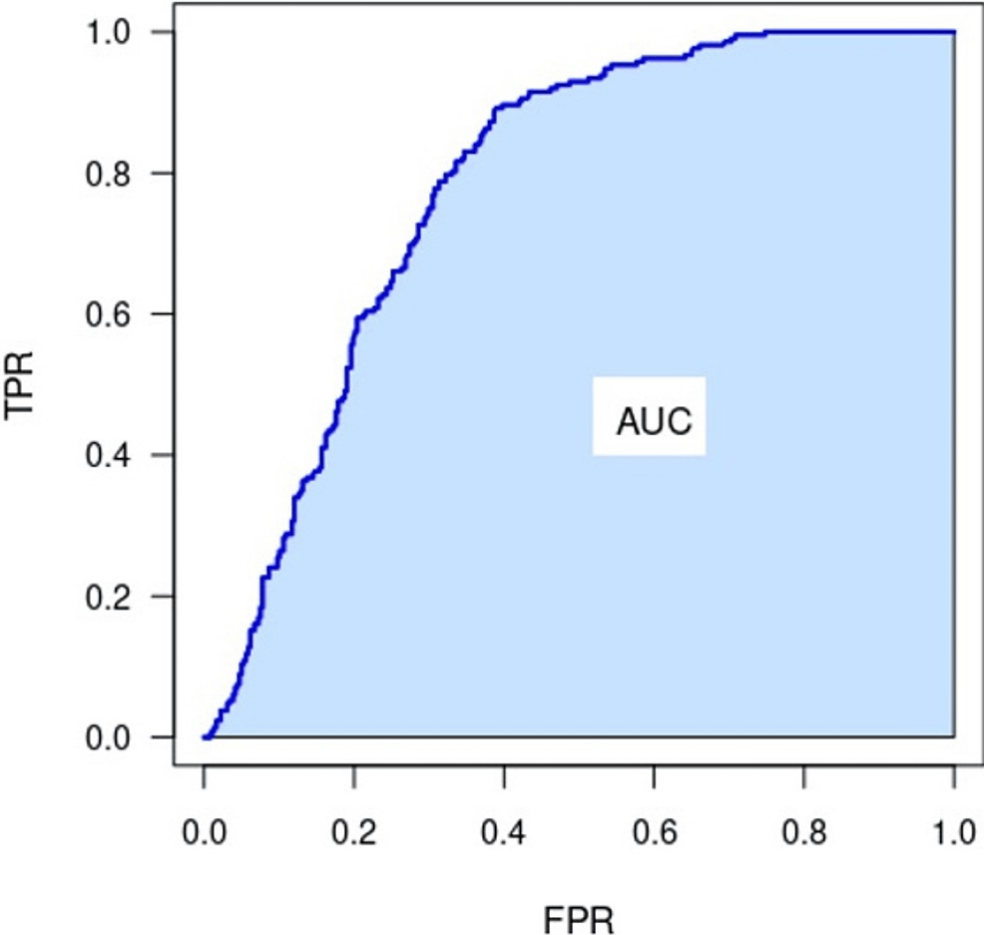
The area under an ROC curve. The area under a curve refers to the two-dimensional space or region that is bounded by the curve itself and the x-axis (or horizontal axis) over a given range or interval. The area under the curved line on the graph represents a measurement or quantity that is accumulated over the x-axis. Image source: ProfGigio, “The area under a ROC curve,” 2022. Accessed via https://commons.wikimedia.org/w/index.php?curid=114508624. CC-BY-SA-4.0. ROC: receiver operating characteristic; TPR: true positive rate; FPR: false positive rate; AUC: area under the curve

**Figure 4:**
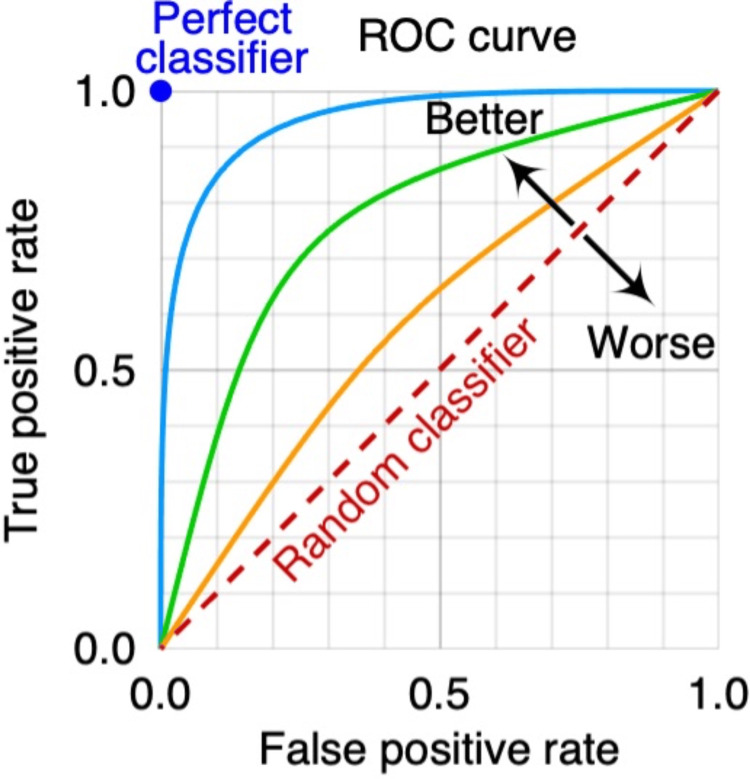
Receiver operating characteristic curve. Receiver operating characteristic curve with the false positive rate and the true positive rate. The diagonal line shows the performance of a random classifier, where the results are no better than random chance. Three example classifiers (blue, orange, green) are shown. Image source: MartinThoma, “ROC Curve,” 2018. Accessed via https://commons.wikimedia.org/wiki/File:Roc-draft-xkcd-style.svg. CC-BY-NC-1.0. ROC: receiver operating characteristic

The traditional calculation of the AUC of the receiver operating characteristic (ROC) curve measures how well the probabilistic model can distinguish between two outcomes: postoperative course with or without complications (Figure 3). The ROC is used to assess the sensitivity and specificity of the model. The ROC plots the true positive rate (sensitivity) against the false positive rate (1 - specificity) at different threshold settings. AUC is a commonly used and well-accepted index of the overall performance of the AI model. For example, an AUC of 0.50 would indicate that the model performed no better than random chance, a coin flip, between the changes of postoperative complication. An AUC of 1.0 means the model discriminates between the two outcomes without errors. Generally, an AOC of 0.7 is considered fair, 0.80 good, and 0.9 or higher excellent. AUC is regarded as a helpful metric because, with it, a single number can be used to summarize the overall performance of an AI model, making it easier to compare different models or assess the impact of changes to the models (Figure 4). However, a high AUC alone does not ensure that AI will necessarily produce a way of distinguishing between clinically useful endpoints nor inevitably produce better clinical outcomes.

Wound Infections

In the United States, surgical wound infections are estimated to cost over 25 billion dollars a year in additional medical expenses. Careful wound surveillance remains a primary means of detecting and intervening in the earliest stages of wound infection. In a joint study undertaken by two independent wound centers at New York University, surgical researchers used 200 photographs of postoperative wound infections to provide a machine learning (ML) database for AI. Errors made by AI in tracing out the extent of wound disruption and infection were compared to the tracings made by knowledgeable surgeons who were blinded to the identity of the patients. There was no significant difference between the performance of AI and that of human surgeons. The study demonstrated that automated wound surveillance by AI was comparable in sensitivity and specificity to surgical experts [10].

Intraoperative Guidance

AI has had some early applications in the operating room. The inroads made by AI include procedure duration prediction, gesture recognition, intraoperative cancer detection, intraoperative video analysis, workflow recognition, an endoscopic guidance system, knot-tying, and automatic registration and tracking of the bone in orthopedic surgery [11]. Human error now represents a more significant source of hazardous errors than technology failures [12]. The operating room has long been seen as the most important source of errors in hospital systems; over half of those errors are deemed avoidable [13]. One of the great limitations in analyzing incident report data from operating rooms is that they function as large data streams from diverse sources. With the advent of AI, there might be a better way to approach, analyze, and measure the data to achieve better outcomes.

Procedural Duration

There have been numerous attempts to apply AI to several different data streams from within the operating room. For example, data extracted from the operative video stream and surgical instrumentation were employed to predict the duration of surgical procedures. Over 80 laparoscopic procedures were reviewed, and both data streams were available for analysis. Combining both yielded an average error of 37% with an average halftime error of 28%, which compared favorably with prior baseline studies, which revealed an average error and average halftime error that both exceeded 128% and were derived from the length of time for which a given procedure had been scheduled to be in the operating room [14]. Many confounding factors affect the predicted duration of a surgical procedure (experience of surgeon and anesthesiologist, type of anesthetic agent used, technical difficulty of the procedure, availability of staff and instrumentation, etc.). A recent analysis of a more homogenous case of ophthalmological surgeries revealed that applying so-called “neuro-fuzzy inference systems” (NFIS) yielded the best predictions about procedural duration. NFIS are systems that combine straightforward neural networks for ML but then incorporate fuzzy logic, which includes intuitive guessing about where the correct answer lies [15].

Gesture Recognition

Because of the ubiquity of computerized devices and communication equipment, there are abundant opportunities for members of the aseptic operative team to inadvertently contaminate themselves by contact with a keyboard or other control surface. For this reason, a group of Korean surgeons decided to create a contactless control surface that depended on hand and finger gestures to control the letter and cursor function. Each team member was allowed to enter up to 30 individualized hand or finger gestures, during which ML occurred. Once there was agreement that adequate training had taken place, the research team investigated the incidence of keyboard contamination. Again, this experiment would have been challenging just a few years ago. However, with AI, entire surgical teams could enter their hand gestures, and there would be clarity between members of the team and problems with retention to reduce inadvertent cross-contamination during operative procedures [16].

Intraoperative Cancer Detection

In oncological surgery, obtaining a gross total or near-total resection is one of the primary objectives of the surgical procedure. One method for the detection of tumor cells in the resection bed is to use Raman spectroscopy to identify cancer cells in vivo. However, with AI, ML can help create better separation between artifact and spectrum indicating in vivo cancer cells. Using AI proved to make the detection of tumor cells 20% more sensitive [17].

Endoscopic Guidance

Videotape collection can be a burdensome problem in the operating room, especially with laparoscopic or endoscopic procedures where a single method can generate hours of videotape. As these videotapes are used both for documentation and training purposes, it often proved difficult to get precisely to the place and the operative frames where discussion could ensue. However, with AI, automatic segmentation of the various lengths and intervals of the videotape was possible, which allowed for a much more efficient way of getting to the points of interest or points of training in the video. AI could differentiate between the various steps in the laparoscopic procedures and automatically bookmark specific segments for review with an overall accuracy of 82% [18]. In a second, unrelated study, ML was employed to tell surgeons which pathway to follow to remove and replace laparoscopic instruments in the field while diminishing the potential for inadvertently damaging tissue or hitting an organ. AI reduced inadvertent instrument collisions by 29% and correctly guessed what instruments would be used [19].

Registering and Tracking Bone

Registration is essential in any computer-assisted orthopedic surgery. The registration of bone from preoperative scans defines the patient’s position and alignment of the operative site concerning the surgical system. Recently, surgeons were able to use AI to preoperatively register the site and position of the patient’s bone so a complete preoperative plan could be correctly aligned with the site. All subsequent steps of the procedure will thus be directly affected by the registration accuracy. With the AI algorithm, there was no need for input from the surgeon. The system showed a weighted pixel accuracy of 98.10 ± 0.99 with a three-dimensional (3D) translational error of 2.75 ± 1.13 mm, which compared very well with intraoperative invasive registration [20].

Intraoperative Guidance During Surgery

AI is now being applied to a myriad of intraoperative guidance systems. For many of these systems, the earliest reports being published are the first applications of AI, and it should be anticipated that the systems will become more robust, precise, and widely deployed in the next few years. AI has been successfully applied to developing a host of guidance system scenarios.

Endovascular Abdominal Aortic Aneurysm

A deep learning model was trained to identify a “Go/No-Go” guidance for determining a “landing zone” during endovascular aneurysm repair (EVAR). The “No-Go” zone was defined by ending up in a position where the stent inadvertently covered or occluded the lowest take-off of the renal artery. The AI model was trained using image sets derived from 110 patients to identify and prevent suboptimal placement of the EVAR. The AI guidance demonstrated a 97% success rate in detecting infringement on the renal arteries [21].

Anatomical Recognition in Association With Laparoscopic Cholecystectomy

One of the most common errors that can occur during an inoperative procedure is a mistake in visual perception or an outright misidentification, leading to an error in judgment and a misinterpretation of an enemy. In this regard, AI has proven helpful by providing enough deep learning to assist surgeons with real-time anatomic guidance.

In one study, AI models were trained on over 2,600 random frames from 290 laparoscopic cholecystectomy videos. These were procured from over 37 countries, 136 institutions, and 153 expert surgeons. AI was used to identify anatomy within the surgical field. The study’s primary outcomes were intersection-over-union (IOU) and an F1 score, which were validated spatial correlation indices. The mean IOU for identification of the liver was 0.86 ± 0.12, gallbladder was 0.72 ± 0.19, and hepatocystic triangle was 0.65 ± 0.22. AI proved helpful in identifying anatomic landmarks during laparoscopic surgery [22].

Parathyroid Gland Detection During Thyroidectomy

One of the critical steps in any thyroidectomy procedure is to correctly identify and spare the parathyroid glands. Particularly in an intraoperative situation, this can often prove quite tricky. Developing an AI model for determining the parathyroid glands could reduce the procedure’s morbidity. Video clips of parathyroid glands were collected during routine thyroid lobectomy procedures. The images of the parathyroid glands were confirmed. Then, they were used to form three types of data sets according to augmentation status, namely, baseline, geometric transformation, and generative adversarial network-based image in-painting. The AI model could correctly identify parathyroid glands on the baseline data 77% of the time. However, AI performance was further enhanced by applying geometric transformation and image in-painting augmentation methods. The geometric transformation data and augmentation data sets between these two modalities proved superior to the image in-painting AI model. The average precision was 79% in the transformation dataset vs. 78.6% in the inpainting dataset. However, the model was then subjected to images of utterly different thyroidectomy approaches, and imaging in-painting proved to be the most effective method for visualizing the parathyroid glands with AI; hence, using augmentation methods holds excellent promise down the road for enhanced AI identification and guidance applications [23].

AI Intraoperative Guidance for Glioma Resections

Malignant gliomas are one of the most common intracranial tumors, and surgical resection is a part of the standard treatment. Because gliomas can be diffusely infiltrative, resectioning them from the brain parenchyma can be challenging. To enhance the tumor resection, 108 patients with gliomas were imaged with MRI and then selected and divided into intraoperative magnetic resonance-assisted glioma section and a control group where the conventional surgical resection was performed without MRI guidance. After the tumor section, the patients were evaluated using the National Institute of Health Stroke Scale (NIHSS) score, a Karnovsky score (another measure of neurological function), and postoperative intracranial infection surveillance. The results indicated that AI-MRI intraoperative guidance dramatically improved the average tumor resection over the control group (p < 0.05). There was no significant difference in the Karnovsky scores, NIHSS scores, or infection rates between the two groups. The study clearly showed that using AI-MRI interoperative guidance improved tumor resection [24].

Guidance Identifying Liver Vessels During Laparoscopic Liver Resection

The recognition of liver vessels during a parenchymal resection of the liver is a crucial part of the surgical technique for laparoscopic liver resection. In one study, an AI model was developed to help recognize hepatic veins and Glissonean pedicles in the liver. In total, 2,421 frames were extracted from 48 laparoscopic liver resection video clips. The AI model’s false negative, false positive, and vessel differentiation ratings averaged 4.36, 3.44, and 3.28, respectively, on a five-point scale. Studies suggested that AI guidance would benefit minimally invasive surgery and surgical liver resection [25].

Augmented Reality Image Overlay

Augmented reality-based image overlay (ARIO) refers to an image of the anatomy depiction that is intraoperatively projected onto the operative field, specific to an individual patient. It has been, in many regards, every surgeon’s secret dream. Unfortunately, it has been largely unrealized because of the sheer size of the databases and the demands of co-registering the patient’s preoperative imaging data with the patient’s pertinent anatomy. Not only must that data set consider how the patient is positioned on the table, but it must also change the surgeon’s head position, as this also changes the point of view by which the overlaid anatomy will be displayed. However, with the advent of access to greater computer storage and processing speed via cloud-based servers and new, better models for imaging such as inpainting, Adaptive Neuro-Fuzzy Inference System (ANFIS), Neural Radiance Fields (NeRF), or Gaussian splatting techniques, rapid and seamless image reconstruction is now possible. The net effect is to make what was once an imaging trick only achievable with supercomputers and place it within the reach of any operator with just a moderately well-powered laptop [26]. So-called “tracking systems” permit the patient’s anatomy to be “matched” to the radiographic database. These include tracking systems that use implantable osseous registration marks, simple shape co-registration (e.g., dental registration), optical tracking, and infrared tracking [27]. A whole host of factors impact not just the ease of co-registration in the operating room but also directly impact the accuracy of that registration during colonoscopy to be able to sample and biopsy. While with indwelling osseous markers, co-registration accuracy can approach a submillimeter limit, in general, the level of accurate localization of five millimeters [28].

ARIO guidance systems have been applied in a host of settings. For example, AI overlays have helped colorectal surgeons locate suspicious lesions on colonoscopy and helped reduce the number of missed lesions [29]. Similarly, AI imaging strategies help identify suspicious, infiltrative lesions transmurally [30]. AI-driven imaging overlays helped improve the percentages of gross total resections [31]. In neurosurgery, AR-related guidance systems allow surgeons to achieve more complete resection of intraparenchymal neoplasms [32]. In orthopedics, ARIO guidance has helped reduce intraoperative time and improve outcomes [33]. Finally, in robotic surgery, ARIO has helped reduce the number of closed or “keyhole” approaches that have had to be converted to open procedures, reduced collateral muscle and skeletal injuries, reduced the need for ventilatory support, and unintended collateral surgical injury [34]. In a similar but unrelated study, 50 patients with gliomas in the insular region of the brain were divided into a control group, which underwent standard resection, and an experimental group, which used a form of AI-MRI intraoperative guidance. Again, the median extent of resection of the tumor mass increased significantly from 79% (range = 58% to 98%) when MRI guidance was used. The p-value for the experimental group versus the control group was less than 0.001. In addition, the Karnofsky performance status was significantly higher in the experimental group than in the control group at three months after surgery, and the median progression-free survival of the AI-MRI-assisted group was 18 months (range = 9 to 42 months) versus the control group at 15 months (range = 3-32 months). The p-value was less than 0.01. Finally, the median overall survival of the AI-MRI-assisted group was 28 months (range = 14 to 49 months) compared to the control group which was 18 months (range = 77 to 38 months), with a p-value <0.035 [35].

AI Guidance in Vertebroplasty

Vertebroplasty, a minimally invasive spinal surgery, can be a challenging procedure due to the distortion of the anatomy caused by fractures or tumor infiltration. Surgeons have traditionally relied on two-dimensional (2D) fluoroscopic imaging for intraoperative guidance, but this technique has its limitations, particularly in terms of patient radiation exposure. However, a recent study found that augmented reality (AR) combined with AI could provide a novel and effective navigational technique. This technique superimposes virtual 3D anatomic data onto real-time visual images, resulting in a fused AR image that improves accuracy and safety during the procedure. The authors of the study found that the AR-AI-guided percutaneous vertebroplasty technique was not only technically feasible but also resulted in significantly lower patient radiation exposure compared to the standard fluoroscopically-guided group. The reduced dose-area product of 182.6 ± 106.7 milli-gray per centimeter squared and 5.2 ± 2.6 seconds of fluoroscopy time versus 367.8 ± 184.7 milli-gray per centimeter squared and 10.4 ± 4.1 seconds for the control group, respectively, demonstrates the effectiveness of this technique. This study’s findings suggest that AR-AI-guided percutaneous vertebroplasty is a promising technique that can improve the accuracy and safety of the procedure while reducing patient radiation exposure compared to the conventional fluoroscopic guidance technique [36].

AI and Surgical Technical Assessment

AI has been increasingly applied to assess surgical techniques. To determine the proficiency of surgical trainees quantitatively and to ascertain their progress in a rigorous, automated fashion free from the risk of supervisory bias and interrater variability offers postgraduate training programs a powerful new tool for assessment. Intraoperative assessment is, as one surgeon put it, “blunt.” Inconsistent observation, data gleaned from a select family of index cases, and postoperative statistics such as length of hospital stay, morbidity, and mortality are, at best, crude and indirect indices of a trainee’s acquisition of surgical experience. Many surgical educators have developed a host of standardized scales or inventories to quantify the technical expertise of surgical trainees. They have included validated measures such as Objective Structures Assessment of Technical Skills (OSATs), developed at the University of Toronto in the 1990s as a two-part assessment of surgical trainees [37]. It provides a well-developed, standardized checklist of essential steps and milestones in common procedures. This checklist provides a global rating scale of generalized surgical skills. The OSATs have been validated both for intraoperative procedures [38] and bench-side training procedures [39].

However, the OSATs are not a panacea. The standardized assessment has been criticized for evaluator bias unless precautions are taken to ensure that the surgeons doing the evaluations do not personally know the trainees. In addition, issues have been raised about excessive variance related to the experience and seniority of the surgical trainee, an inability to assess surgical “judgment” in the face of a surgical emergency or intraoperative deterioration, a lack of assessment of interprofessional behavior and teamwork skills, and, finally, a significant financial investment in equipment, disposable goods, and faculty time [40].

Similarly, surgical educators in the OB-GYN residency at the University of Washington created a seven-station evaluation of all residents in their training program. The research used various procedures performed on anesthetized pigs, including four standard laparoscopic procedures and three open surgical procedures. Each resident was evaluated using three separate scoring systems at each surgical station. The scoring systems included a procedure-specific checklist, global technical skill reading scales, and simple pass/fail evaluation for each task. The three systems were compared for construct validity. The international rating system and task-specific checklists exhibited high reliability in stratifying residents by experience and expertise. Combined systems (i.e., checklist and global) also seemed capable of identifying trainees requiring additional training and mentoring to raise their skill levels [41]. The researchers and surgeons involved in the study took much time, care, and expense to provide a comprehensive surgical evaluation. When carrying out the study, they reported that only 17% of the OB-GYN programs in the country deployed objective assessments in evaluating their residents. In contrast, a full one-fourth of all postgraduate training programs had no objective evaluation at all [42].

A staggeringly wide array of tests, assessments, scales, and inventories have been evaluated in the hopes of accurately predicting which individuals possess the correct combination of psychomotor skills to become good surgeons. These evaluations included outright neuropsychological testing, the Minnesota paper form test (a test aimed at predicting manual dexterity), the Purdue pegboard test, as well as tests of visual-spatial assessment (e.g., hidden figures test). The University of Toronto has been a leading center for surgical education research. In a review paper [43] evaluating various measures of technical surgical proficiency, the author pointed out that three major questions needed to be addressed in assessing the over-arching question of surgical training. The first was to determine if there was a problem (or, at least, an “inefficiency”) in teaching adequate technical skills. The second question was to assess if the current surgical teaching methods were turning out excellent surgeons. Finally, the third question was whether the surgical faculty perceived their responsibility to transmit technical expertise as part of their oversight.

There is no question that teaching surgical proficiency has become more complex. One reason is that there are increasing restrictions on how long residents may remain on duty, resulting in fewer hours in the hospital and the operating room. That also means less time in residency training available for practice and contact with patients and faculty. It is also recognized that the medicolegal environment has undergone a sea change in the last several decades as both government and educational authorities have enforced a much closer supervisory oversight in the operating room. Regulations (e.g., the government-administered Medicare) that urged surgical faculty to make their physical appearance in the operating room more evident and exert more responsibility for the critical portions of surgical procedures than in the past also translated into trainees needing more direct surgical responsibility in the operating room. This mandate for clinical accountability meant less surgical independence and less opportunity for trainees to exert their surgical judgment without consultation. In addition, as greater efficiencies were imposed on hospital-based operations, patients moved through the hospital system more quickly than 50 years ago. In so doing, the residents have far fewer opportunities to observe patients longitudinally, in serial fashion, as they proceed from the clinic and preoperative assessment through surgery and then on to the postoperative period and recovery. These factors negatively affect the scope of resident education and even patient outcomes [44].

In the final analysis, the thread of surgical continuity is much more challenging to preserve in patients now than in years past. A half-century ago, Lippert et al. astutely pointed out that the notion of a surgical trainee merely needing to acquire a specific set of psychomotor skills was an oversimplification [45]. Their argument illuminates the fact that the acquisition of surgical techniques is a multifaceted process, advancing not only with muscle memory and practice but also with a certain amount of intellectual growth and maturational insight.

Surgical researchers at Columbia University used the Global Operative Assessment of Laparoscopic Skills (GOALS) and it has been shown to have construct validity in the context of evaluating the performance of surgical residents as they performed a laparoscopic removal of a gallbladder (i.e., a cholecystectomy). GOALS was developed by Melina Vassiliou and colleagues at Columbia University as an outgrowth of the earlier work they had done on upper esophagogastroduodenoscopy (EGD) and colonoscopy. The GOALS proved an easy tool to use during simulated bench-style dissection but required endoscopists who were both well-versed in their specialty and well-trained in the uniform application of rating criteria if interrater variability was not to overwhelm the value of the test [46,47].

As ML began to be applied to surgical performance assessment, it brought with it a promising prospect, the hope that quantifiable objective evaluation could be achieved while still preserving construct validity. ML also held out the possibility that the assessment could be carried out without bias, the results would be available almost immediately, and the testing could be administered without any need for a human instructor or proctor to be present. Several different styles of ML have been applied to surgical assessment with the most important ones being hidden Markov models (HMMs), support vector machines (SVMs), and artificial neural networks (ANNs) [48].

HMMs are useful for modeling sequential data, such as the movements and actions performed during surgery. They can capture the underlying states (e.g., surgical steps) and transitions between these states based on the observed data (e.g., tool movements, video frames). HHMs are useful for modeling complex, non-linear relationships in sequential data and providing quantifiable insights into discrete steps of a surgical procedure [49]. SVMs are useful for classifying surgical skill levels based on features extracted from surgical data (e.g., tool movements and video frames). They can model non-linear decision boundaries and are effective with high-dimensional data, meaning data spread out across many columns and dimensions [50]. ANNs are a class of ML models inspired by the brain’s neural networks. ANNs are composed of interconnected nodes that can learn to perform tasks by considering examples without explicit programming. ANNs can achieve high accuracy with large training databases [51]. In brief, for a given surgical procedure, HMMs would excel at developing the sequential steps of the procedure, while SVMs would be well suited to determining the level of proficiency exhibited by various candidates. SVMs excel at providing algorithms for evaluating surgical procedures, while ANNs (especially, convolutional neural networks) can learn from the extraction of raw surgical data (e.g., video) and then accurately map that data back to the discrete procedural steps for classification.

One of the great advantages of employing such ML is that one can feed in a series of videotapes and allow the agent sufficient time and enough iterations of the procedures on the video to begin to identify critical milestones of the procedure. It can produce a checklist and evaluate how well the trainee can carry out the procedure without incurring the problems of biased observers [52]. ML can also combine its checklist with other databases. For example, they are carrying out a robotic surgical procedure (like with the Da Vinci system; Intuitive Surgical, Sunnyvale, CA) that allows ML to feed in additional kinetic data streams from the robot’s articulated arms, or derived from sensor systems embedded in the instrumentation, or even worn on the surgeon’s extremities to accelerate the development of better-pooled data for technical assessment [53]. In addition, there is a need for a finer, more granular range of skills assessment so that proficiency can be broken down into a more graduated performance than, say, a simple binary classification, such as novice or expert [54].

The Holy Grail of surgical proficiency is to develop a thorough, sensitive, accurate method of evaluation using AI that can assess a surgeon’s technical proficiency with sufficient speed and sensitivity capable of stratifying the effects of experience, practice, and facility with a procedure. The feedback should be free of the distortions introduced by including different evaluators, too many procedural differences, alternate venues (e.g., bench surgery versus operating room), varied settings (human, animal, or simulated tissue), different approaches (open procedure versus minimally invasive), and, finally, the actual measures being collected. Standardization needs to be improved, and the possibility of comparing it with the experiential framework of a single individual, between individuals in a given program, or between training programs is fraught with methodological shortcomings [55].

One double-blinded randomized controlled study evaluated a group of novice trainees who were asked to practice fundamental laparoscopic skills suturing skills (FLS) until they were deemed to have reached an expert level of proficiency. In the study, novices were randomized into four groups concerning the timing and quality of the feedback they received when they made errors. The group that received both “instant” buzzer (audio) feedback and verbal “error” feedback from an examiner each time they made a mistake performed significantly better on a laparoscopic fundoplication than any other group. This “instant feedback” group' achieved proficiency in half the trials required by the delayed group and received higher scores in the operating room where they performed the fundoplication procedure. This group was compared to the other groups, including those that received only delayed feedback. Their enhanced performance indicates that combining multiple modalities of feedback tightly coupled to the commission of errors can improve psychomotor skill acquisition [56].

AI can be used to directly access video footage both as a way of evaluating procedural proficiency and as a method for analyzing variations in surgical techniques and how they relate to surgical outcomes [57]. One study compared the analysis performed by AI to that of expert surgical raters (using the Global Evaluation Assessment of Robotic Skills, or GEARS), looking at nearly 100 videotaped segments of peritoneal closures. There was an excellent correlation between what expert raters labeled technical efficiency and what the AI algorithms had isolated as observed tool movement. Human analysts using GEARS assessed bimanual dexterity, and these measures correlated closely with the AI-derived measures of bimanual (or simultaneous) instrument movement. The study demonstrated an excellent correspondence between AI-derived parameters and the validated measures used by trained, expert evaluators [53].

As much of the material in a hospital’s procedural databases is in the form of intraoperative video, all of it might, one day, be subject to routine AI-derived evaluation. It would yield exciting insights concerning anatomic variants and routine “squeeze points” in the procedure where direct line-of-sight visualization, exposure, or specific maneuvers offer procedural challenges or insights into best practices [58]. This material could be made available for AI analysis in a timely fashion if there were technical errors or concerns about procedural proficiency for granting specific credentialing. That said, there are deep-seated concerns in the surgical community about the potential for abuses of unauthorized access and violations of privacy. Furthermore, there is growing recognition that perfunctory, routine review of all videotape footage produced in the operating room suites could lead to mandated archiving of all surgical footage. It is feared that it might create new opportunities for medicolegal challenges [59].

AI for Ultrasound Guidance for Delivery of Regional Anesthesia

Often, regional anesthetic is delivered under ultrasound guidance for a period; however, interpretation of the anatomy can be difficult not only because the anatomic structures themselves are distorted in an arcuate fashion (because of the head of the ultrasound device) but also because the anesthesiologist must also manipulate a needle in the field at the same time as obtaining the ultrasound. In one study, the authors used AI to help identify critical anatomic features to facilitate the delivery of ultrasound-guided regional anesthetic. In this case, the authors also used a heads-up display, which assisted the user in visualizing the anatomy while still having their hands free to operate the ultrasound head and the needle without having to look over at a screen and look away from the field. B-mode ultrasound images (video) were labeled, and the frames were then labeled and used to train ML algorithms to provide associations between the labeling anatomy and the underlying structures. The anesthesiologists found the AI-enhanced ultrasound anatomy helpful for creating landmarks for the anesthetic delivery [60].

AI has been shown to generate beneficial virtual models of the patient’s anatomy for preoperative imaging and patient-specific data. Various orthopedic conditions (congenital, trauma, acquired, inflammatory) can be categorized and described using AI systems. ML can then recognize certain features and patterns in medical imaging, enabling orthopedic surgeons to decide on the most useful and suitable approaches. In orthopedic surgery, AI imaging combines several modalities, including traditional X-rays, CT scans, and MRI scans. During surgery, AI has proven helpful for navigation and guidance. Specifically, AI has been very useful in guiding the sizing and accurate placement of bony implants for joint replacement and guaranteeing ideal alignment. The degree of accuracy AI provides surpasses that of unassisted human error. In addition, AI has also been found to help evaluate postoperative risks of infection and guide rehabilitation and postoperative care by using wearable technologies and machine learning to help deliver personalized therapy and remote monitoring of patients’ feelings and progression [61].

AI Guidance in Orthopedic Surgery

AI algorithms have been successfully applied to the detection and localization of tumors in the proximal femur. In a study, over five hundred radiographs of the femoral head were used to train a convolutional neural network (CNN) model for this task. The dataset included 94 radiographs with malignant neoplasms, 120 with benign neoplasms, and the remainder were normal. The resulting model achieved an impressive area under the receiver operating characteristic curve (AUROC) of 0.953 (95% CI = 0.926-0.980). Furthermore, the diagnostic accuracy of the CNN (0.853) was significantly higher than that of four subject matter experts who were asked to evaluate the same films (0.794) (p = 0.001). The mean sensitivity, specificity, precision, and F1 score of the CNN model were 0.822, 0.912, 0.829, and 0.822, respectively, whereas the mean values for the four doctors were 0.751, 0.889, 0.762, and 0.797, respectively [62]. These results demonstrate the utility of AI as an adjunct tool for evaluating radiographs of the femoral head.

A similar approach has been applied to the evaluation of soft tissue knee injuries. Researchers developed a CNN algorithm designed to identify tibial plateau fractures with adjacent meniscal defects on MRI scans. When compared to arthroscopic findings, the algorithm achieved a sensitivity of 69.9%, a specificity of 93.2%, and an accuracy of 95.3%. This study documented the correspondence between preoperative AI-based diagnoses and eventual intraoperative findings [63].

A recent meta-review investigated the use of AI in 12 studies analyzing over 300,000 postoperative radiographs following total hip arthroplasty (THA) and total knee arthroplasty. The combined AI-based implant identification achieved an AUC ranging from 0.992 to 1.0. The AI models were also able to predict the risk of dislocation post-arthroplasty, with an AUC of 76.67 over 8,500 training radiographs. Additionally, the model was asked to identify the loosening of THA implants with 88.3% accuracy using 420 training radiographs. The review concluded that there is great promise in using AI for postoperative evaluation, especially with regards to THA but noted slight methodological differences between the studies and called for a more robust, universal algorithm to be applied across multiple institutions in the future [64].

Multimodal Molecular Imaging for Robotic Surgery

In a fascinating and challenging application of AI for intraoperative guidance, a European consortium developed techniques whereby radionuclides could be delivered to a tumor bed (in this case, esophageal cancer). This so-called “molecular imaging” essentially used low-level radiation in the tumor target to assist a da Vinci robot with intraoperative localization. In addition, molecular imaging was combined with CT, MRI, and intraoperative ultrasound to yield a potent combination of radio localization and tumor projection. This multimodal imaging yielded a potent and innovative anatomic overlay to guide surgeons in their tumor resection using a robotic periscopic technique (that allows a true stereoscopic 3D image of the operative field). This combined intraoperative imaging using a molecular approach with a radioactive tracer, magnetic imaging, and intraoperative ultrasound demonstrates how AI can be used to coordinate multiple imaging modalities [65]. This preliminary study and proof of concept suggest that integrating molecular imaging might bring precision surgery to a new level that combines nuclear medicine with computer-assisted diagnostic planning and robotic navigation and detection.

Intraoperative Decision Support

There are innumerable ways in which AI may be applied “extra-operatively,” that is, outside the context of the actual surgical therapy delivered to the patient on the operating table. Dr. Jennifer Eckhoff, an AI and Innovation Fellow at the Massachusetts General Hospital, has summed up what she believes will be the “killer app” of ML in surgery thus: “Simultaneously processing vast amounts of multi-modal data, particularly imaging data, and incorporating diverse surgical expertise will be the number one benefit that AI brings to medicine” [66]. In particular, surgeons will be able to cull out not just the perils in general, the risk calculated through the numeric spread of the herd, but instead drill down on the unique, personal factors that define risk and benefit for their patient. Through the hitherto unimaginable calculations of the millions of cases, AI can help us arrive at the mathematical delineation of a single individual, the exceptional circumstances that may give them an edge at responding better to surgery, or the personal weaknesses that might inevitably bring disaster. Surgery has always been a game of numbers, but we have never seen numbers with such breathtaking clarity. There are AI models that can tell the surgeon how likely it is that the patient may succumb to sepsis [67], the potential that this patient will wean quickly off on a ventilator in the ICU [68], the odds of survival [54], and the likelihood the patient will not live [69].

However, AI alters everything it focuses on. It is not just in assessing the patient for whom changes will be wrought. AI will help evaluate who might make the better transplant candidate [70], how best to assign operating room block time to derive higher productivity from the operating rooms [71], better anticipate bed availability [72], and schedule staff [73] and surgical inventory [74] to extract additional efficiency and quicker room turnover [75]. AI will also be able to handpick the best surgeons for each kind of procedure, the ones with the greatest experience, the shrewdest judgment, and the best outcomes [76]. 

We will all be facing a brave new world for sure.

Future: going into the unknown, doing the inconceivable

“This, therefore, is a faded dream of the time when I went down into the dust and noise of the Eastern market-place, and with my brain and muscles, with sweat and constant thinking, made others see my visions coming true. Those who dream by night in the dusty recesses of their minds wake in the day to find that all was vanity; but the dreamers of the day are dangerous men, for they may act their dream with open eyes, and make it possible” [77].

Many have pointed to the decades straddling the middle of the twentieth century as the “golden age of Medicine” [78]. The field of medicine had reached new heights of popularity in the eyes of the public, riding atop a wave of dizzying accomplishments that included a host of new vaccines, a vast array of new antibiotic therapies, the first heart-lung bypass, and the earliest inroads into organ transplantation. It reached its apogee with the introduction of the Salk vaccine, which finally began to end polio. But many see the end of the first half of the twenty-first century as holding the potential for becoming a second Golden Age because of the arrival of AI on the scene because of its ability to propel the advent of mRNA vaccines (that were first deployed in the COVID-19 pandemic), remote wearable health monitors, breakthroughs in preventive health maintenance, and enhanced chemotherapy and immunotherapy for systemic cancer. Amid all these factors, none is as much of a game-changer as the injection of AI into medical education and healthcare management. It promises more comprehensive access to many segments of the population who have had difficulty being embraced by the medical system. In an economy where healthcare costs soar and medical care and insurance represent almost one-fifth of the American economy, AI promises to bring economies of scale and enhanced efficiency that could amount to savings of up to 10%, roughly $200 billion per year [79].

Keeping up with all the areas within the disparate medical subspecialties where AI has made stunning progress is challenging. The pace of AI’s ascendancy has been swift, especially in areas of medicine where imaging is the central focus of much of what the healthcare professional provides; this includes pathology, radiology, dermatology, and ophthalmology. Some have estimated that there is a 50% chance or better that AI will outperform physicians (and surgeons) by 2050 [80]. In many cases, this improvement will represent nothing more than refinements of what we have seen trending in the last few years from the introduction of AI within healthcare.

Miniaturization of Surgical Instrumentation

What changes AI will bring to the field of surgery in the not-too-distant future almost makes the rest of the alterations in healthcare appear muted and pale by comparison. Experts expect significant changes in three areas: the miniaturization of surgical instrumentation and micro-robotics. Both laparoscopic and robotic technologies have made enormous inroads in miniaturization because they represent minimally invasive technologies. Both kinds of technologies will gain significant advantages as surgical instrumentation shrinks in size. The trend in miniaturization responds to (a) a drive to reduce the instrument’s volume balanced against (b) a sizeable dexterous workspace [81]. Much of the minimally invasive instrumentation requires a tether or linkage to operate the instrument. To allow for further miniaturization, engineers must design more sophisticated tetherless instruments. This freedom from a tether will enable instruments to be introduced into the body through a conduit. This route would allow the instrument to travel into the organ or be delivered nearby. The control over such instruments would likely be electromagnetic so that they can operate at a distance. The general idea would be that the patient would “swallow the surgical instrumentation,” or, in the case of a cardiovascular instrument, it would be delivered by injection [82]. Small microgrippers (1.5 mm diameter) have been built that can be injected into a bovine bladder, travel to a site in the bladder, obtain a tissue biopsy there, and then return with a suitable amount of specimen in its jaw [83]. Deploying swarms of such miniaturized surgical instrumentation would quickly overwhelm a human surgeon’s ability to track dozens of such instruments so that task would have to be managed with the help of AI. Similarly, miniaturized instrumentation is also foreseen when creating miniaturized robotic platforms for natural orifice transluminal endoscopy [84].

Globalization of Surgical Education

The COVID-19 pandemic pressured academic medical departments to develop the infrastructure and assets to allow students to study and attend classes remotely. There is a need for more qualified healthcare practitioners worldwide. Even medically advanced countries have increasingly begun looking to the global marketplace to recruit staff to meet their needs [85]. New technologies such as AR virtual reality and simulation platforms have made collaborative international training more accessible. Furthermore, the interest in access to global surgical education is mutual. Not only are the leading economies in the world reaching out to low and middle-income countries (LMICs) but there is also interest in these host countries to develop a more accessible global surgical education. The shortage of surgeons in the United States continues to increase, and the current expectation is that 25% of the surgical workforce in the United States will be opposed to being composed of international medical graduates [86].

Robotic Hybrid and Autonomous Robots

Numerous avenues of robotic research with AI are currently being pursued. Most of these efforts involve robotic assistants or surgical robots (such as da Vinci) in the operating room. There are compelling reasons to pursue the development of autonomous surgical robots. The first is that robots can perform surgical tasks with more precision and accuracy than humans. Second, surgical robots may help reduce surgeon fatigue and burnout and the concomitant errors that arise from these two conditions. Autonomous robotic systems are uniquely suited to perform repetitive and delicate tasks without physical limitations and without the potential for human error that surgeons face, especially during lengthy procedures. The third point is that autonomous surgical robots would provide expanded access to surgical care. Autonomous robots will have the potential to perform complex surgical procedures where human surgical expertise is unavailable, or coverage is vastly inadequate. As there is a limitation on the number of trained human surgeons available, the development of fully autonomous surgical robots will offer substantial relief. Robotic surgery is heavily weighted toward minimally invasive surgery, and most robotic operations produce smaller incisions and more rapid recovery times. This trend should continue as autonomous surgical robots are refined and perfected. Finally, we can expect autonomous surgical robots to be constantly improving. Given ML models, robots would continually be learning from their past surgical experiences, and there is the potential that they could be pooling their shared surgical experiences through mutual linkages. Finally, there is recognition that there are unique environments to which human surgeons are poorly suited. The three main areas where this is most relevant include the battlefield, disaster areas, and extraterrestrial space travel.

It is recognized that autonomous surgical robots could provide critical surgical care in hard-to-reach areas and perform procedures with great precision without human supervision or intervention. While telesurgery is feasible in many scenarios, circumstances can arise where a human surgeon operating a “surgical drone robot” from a distance will not work. The first issue is latency, which means there can be a significant delay or lag between when the human operator issues a surgical command and when the transmitted command is transmitted to the robot so it can carry out the order. Second, communications are notoriously susceptible to disruption in isolated, environmentally hazardous conditions or on the battlefield. In short, someone would have to face a situation in which telesurgery is being carried out at a distance, and then there is an interruption of the connection between the human surgeon and the drone robot. A completely autonomous robot would be far more desirable under such conditions. Finally, such a telesurgery system would also have to be carefully protected because it would be susceptible to hacks, which would be extraordinarily disrupted. Indeed, roving robotic medics would be a welcome addition to the battlefield. In addition, such medics could be designed and constructed in such a way as to be able to carry injured soldiers to safety for other medical evacuation or a disposition to a forward battalion field station. While such telepresence systems are becoming more accessible to design and produce, they still have not undergone sufficient rigorous testing to be deployed to handle wounded personnel in the field.

However, there is one environment where there is now gradual recognition that autonomous surgical robots will provide the only reasonable long-term solution: space travel. First, there needs to be a density of individuals in space to mandate the placement of a full-time surgeon in residence. Second, the only surgeons who could fill that role would be individuals who were also fully trained for the rigors of space missions. A small habitat station on the moon would still permit some potential for telesurgery because any signal latency between a surgical operator on Earth and a drone robot on the moon is small. The distance between the Earth and the moon is only 385,000 km, which puts the latency at approximately 1.3 seconds. However, by contrast, a mission to Mars provides different conditions. The distance between Earth and Mars is between 78 million and 378,000,000 km, depending on their relative orbital positions. That would put the latency between transmission on Earth and reception of the signals in the space station on Mars between 4.3 minutes and 24 minutes (when the planets would find themselves on opposite sides of the sun in their respective orbits). This latency is far too long to permit any meaningful telepresence. For this reason, autonomous robots are the most likely alternative solution.

At a minimum, it is recognized that autonomous surgical robots must perform a particular constellation of surgical procedures during space travel. These would include treating traumatic injuries, which could undoubtedly occur during space missions and could be sustained during extravehicular activities or accidents. Trauma would presumably run the gamut from penetrating injuries to fractures and internal bleeding. In addition, autonomous surgical robots would need to be able to perform emergency surgeries for acute conditions such as appendicitis and cholecystitis, which can occur without prodrome and warning period. In these situations, delayed treatment until return to Earth might not be possible and could represent a life-threatening situation. There are at least, to date, two reports of suspected appendicitis in Russian cosmonauts, one of which resulted in an emergency return to Earth. There can also be referred symptoms of urolithiasis and or prostatitis, which mimic those of appendicitis [87]. Given the incidence and frequency of appendicitis and cholelithiasis in age groups, they are likely to overlap substantially with those of astronauts. It has been suggested that potential candidates for prolonged space travel undergo prophylactic appendectomy and cholecystectomy [88]. In a similar vein, many would suggest that potential astronaut candidates who are going to be assigned to long sustained missions in space undergo MRI cerebral angiography to exclude aneurysmal pathology and diminish the likelihood of intracranial hemorrhagic events in space. Autonomous surgical robots would need to provide wound care and control bleeding in a microgravity environment. Hemostasis is particularly challenging in a zero-G environment where blood and fluids can float away from the surgical site [89]. For this reason, specialized devices and techniques would have to be developed to handle the blood loss in microgravity.

Space offers other unique challenges. For example, because of surface tension issues, fluids tend to pool and form domes that can fragment on disruption by instruments. These fragments can float away and be dispersed throughout the spacecraft, representing a substantial biohazard. Furthermore, the microgravity environment encourages the floating of the bowl so that it abuts the abdominal wall, which would produce a theoretical hazard for instrumenting the peritoneum. One solution that has been proposed is the creation of a hermetically sealed enclosure over the surgical site, and designs would either use pressurized air or sterile fluid as a differential between the anatomic site and cabin atmospheric pressure to prevent evisceration and floating debris [90].

In addition, autonomous surgical robots would likely have to be able to carry out minimally invasive procedures such as laparoscopic or robotic surgery in space. There would be an additional advantage here in that it would keep the surgical field reasonably isolated from the environment of the spacecraft itself. Techniques such as gas insufflation would be needed to maintain proper surgical conditions. When autonomous robots carry out major surgical procedures, there is a need to administer anesthetics and maintain patient stability during the procedure. A combination of local and intravenous anesthetic would be preferred over general; however, it is recognized that general anesthetic might also need to be utilized. Finally, autonomous surgical robots would have to be able to prevent contamination of the surgical field and the spacecraft from microbes in a particulate manner. Therefore, specific sterile techniques and containment systems for deep space would be critical.

Several factors continue to drive up the cost of healthcare delivery, including an aging population with increasing medical needs, rising frequency of accessing and utilizing healthcare services, and the development and proliferation of advanced new technologies. However, much of these expenditures can be traced to outright waste, inefficient delivery of goods and services, and overtreatment [91,92]. The application of AI in multiple medical models [93] suggests that the integration of AI in the realms of diagnostic algorithms, treatment architectures, programs of risk reduction and prevention, and improvements in scheduling and patient throughput could effectively reduce healthcare spending in the United States alone by as much as 5-10%, representing savings of $200 billion to $360 billion in annual expenditures [79]. Grave concerns about the risks of broadening AI applications include substantial job losses. Estimates in the United Kingdom suggest up to 35% of jobs could be lost over the next 20 years [94]. Additionally, there are clinical data risks, including direct harm from AI errors [95]. Technical risks such as data biases, privacy, and security concerns are also significant [96]. Socio-ethical concerns arise from a lack of transparency [97].

## Conclusions

The world of medicine is constantly evolving, and the integration of AI into surgical therapy is now one of the driving forces behind this transformative progress. As we strive toward a future where AI assumes an increasingly pivotal role in healthcare, it becomes evident that those who are swift to adopt this groundbreaking technology will be the trailblazers in their field. One of the most promising applications of AI in surgery lies in its potential to enhance procedural safety. The precision and accuracy of AI algorithms can empower surgeons to perform even the most complex operations with greater ease and precision, thereby reducing the risk of complications and improving patient outcomes.

The benefits of AI extend far beyond the confines of the operating theater. By meticulously monitoring patients during their postoperative recovery, AI systems can detect early warning signs of complications, enabling healthcare providers to intervene promptly and mitigate the development of serious issues. Admittedly, change is seldom an effortless endeavor, and many individuals may understandably harbor reservations about embracing new technologies. However, the substantial potential advantages that AI can bring to surgical therapy and patient care cannot be overstated. We must remain open-minded and receptive to these innovative advancements. Furthermore, the creation of reliable autonomous robots is rapidly becoming a priority in the healthcare sector, both for surgical deployment on the battlefield and in disaster-stricken areas. These robotic marvels possess the capacity to undertake tasks that are too perilous or challenging for human surgeons, potentially saving countless lives in the years to come. Moreover, autonomous surgical robots will be vital in prolonged space travel and extraterrestrial colonization. In conclusion, integrating AI into surgical therapy is an exhilarating and rapidly evolving field, brimming with the potential to revolutionize patient care. The “dreamers of the day” will be the ones willing to embrace the changes wrought by AI and forge a path through the new technological landscape to reach a brighter, safer future for all.
